# Pain Management and Use of Opioids in Pediatric Oncology in India: A Qualitative Approach

**DOI:** 10.1200/JGO.2016.003483

**Published:** 2016-11-02

**Authors:** Paola Angelini, Katherine M. Boydell, Vicky Breakey, Purna A. Kurkure, Marian A. Muckaden, Eric Bouffet, Brijesh Arora

**Affiliations:** **Paola Angelini** and **Eric Bouffet**, The Hospital for Sick Children, Toronto; **Vicky Breakey**, McMaster University, Hamilton, Ontario, Canada; **Katherine M. Boydell**, University of New South Wales, Sydney, New South Wales, Australia; and **Purna A. Kurkure**, **Marian A. Muckaden**, and **Brijesh Arora**, Tata Memorial Hospital, Mumbai, India.

## Abstract

**Purpose:**

Consumption of medical opium for pain relief in India is low, despite the country being one of the main world producers of the substance. We investigated obstacles to opioid use and physician perceptions about optimal pain management in pediatric oncology patients in India.

**Methods:**

Semistructured interviews were conducted with oncologists who work in pediatric oncology settings. A mixed sampling strategy was used, including maximum variation and confirmation and disconfirmation of cases, as well as snowball sampling. Key informants were identified. Interviews were audio recorded, transcribed verbatim, and analyzed by thematic analysis methodology.

**Results:**

Twenty-three interviews were performed across 20 Indian institutions. The main obstacles identified were lack of financial resources, inadequate education of health care providers on pain management, insufficient human resources (particularly lack of dedicated trained oncology nurses), poor access to opioids, and cultural perceptions about pain. Children from rural areas, treated in public hospitals, and from lower socioeconomic classes appear disadvantaged. A significant equality gap exists between public institutions and private institutions, which provide state-of-the-art treatment.

**Conclusion:**

The study illuminates the complexity of pain management in pediatric oncology in India, where financial constraints, lack of education, and poor access to opioids play a dominant role, but lack of awareness and cultural perceptions about pain management among health care providers and parents emerged as important contributing factors. Urgent interventions are needed to optimize care in this vulnerable population.

## INTRODUCTION

Pain affects patients with cancer at all stages of disease and treatment. Pain control is recognized by the World Health Organization as paramount to changing the quality of life of patients and their families.^1^

Cancer prevalence has increased in low-income countries, where 80% of pediatric patients with cancer live.^2^ With increasing improvement in other causes of mortality (eg, infection, malnutrition), the relative burden of pediatric cancer continues to grow.^2^ According to the Central Intelligence Agency,^3^ in 2011, the population of India was 1.2 billion, with almost one third younger than 15 years of age. The overall incidence of cancer is 38 to 124 per million children per year^4,5^ and has increased at a faster rate than that in Western countries in past decades. This rise can be explained by the increased number of cancer registries, improved availability of diagnostic tools, and improved awareness of cancer among physicians, all of which have tackled the problems of underdiagnosis and underascertainment.^4,6^ The estimated actual incidence of pediatric cancer may be even higher than these current figures.

India is one of the main producers of medical opium.^7^ In 1985, the government issued restrictions through the Narcotic Drugs and Psychotropic Substances Act (NDPSA) on opioid prescription and distribution^8^ to prevent their diversion and misuse, which led to a drop in opioid consumption by more than 97%.^7^ Other documented challenges to pain control in India include difficult access to points of distribution and poor education of health care providers in pain management and palliative care.^9-13^ Despite these known issues, few published studies explored the views of medical professionals in pain management, medication prescription, and education^14-16^ and none in pediatric oncology. This article explores the barriers to use of opioids and delivery of optimal pain control to improve the lives of children with cancer in India.

## METHODS

A qualitative approach was adopted that used semistructured interviews (Data Supplement) that allowed us to explore and identify unanticipated problems and select a small subset of physicians as the target for the study to reduce the risk of bias from a low response rate. The research participants were pediatric oncologists who practice in pediatric units in India and were representative of sex, type and place of training (general pediatrics versus pediatric hematology/oncology, Western countries versus India), and location of practice (rural areas versus cities, public versus private hospitals within four states) and included pediatric hematology/oncology, palliative care, surgery, and radiation oncology specialists. A mixed-sampling strategy that included maximum variation and confirmation and disconfirmation cases as well as snowball sampling were used. When available, palliative care specialists were interviewed. Key informants were identified in the host institution. Author P.A. attended the palliative care clinic at Tata Memorial Hospital, Mumbai, India, as well as conducted 1 day of home visits.

The semistructured interview was offered in person after preliminary contact on the phone to explain the purpose and methodology of the interview and to obtain consent. All interviews were conducted by the same interviewer over 1 month, recorded, transcribed verbatim, and analyzed with a thematic analysis methodology. A classic set of coding strategies for qualitative thematic analysis as outlined by Braun and Clarke^17^ was used as follows: Investigators read and reread all transcripts to familiarize themselves with the data and tracked initial and emergent ideas (by using extensive memo writing); relevant features of the data were labeled systematically across the entire data set to generate initial codes; data were collated by relevant code; codes were collated into larger potential groupings, again to gather all the relevant data within a particular theme; groupings were reviewed by checking to see if they were consistent with coded extracts (level 1) and the entire data set (level 2), and thematic maps were generated for analysis; and a written account was produced by selecting compelling extracts and exemplars and by relating the analysis to the research objectives and relevant research literature. The study was approved by the Research Ethics Board of The Hospital for Sick Children, Toronto, Ontario, Canada.

## RESULTS

All 23 physicians contacted agreed to participate in the study (Table 1). Numerous interrelated issues emerged, which were financial issues, lack of education and awareness among health care providers and the general population, lack of human resources, and poor availability of opioids. Their relative importance varied based on geographic area and type of hospital (public versus private).

**Table 1:**
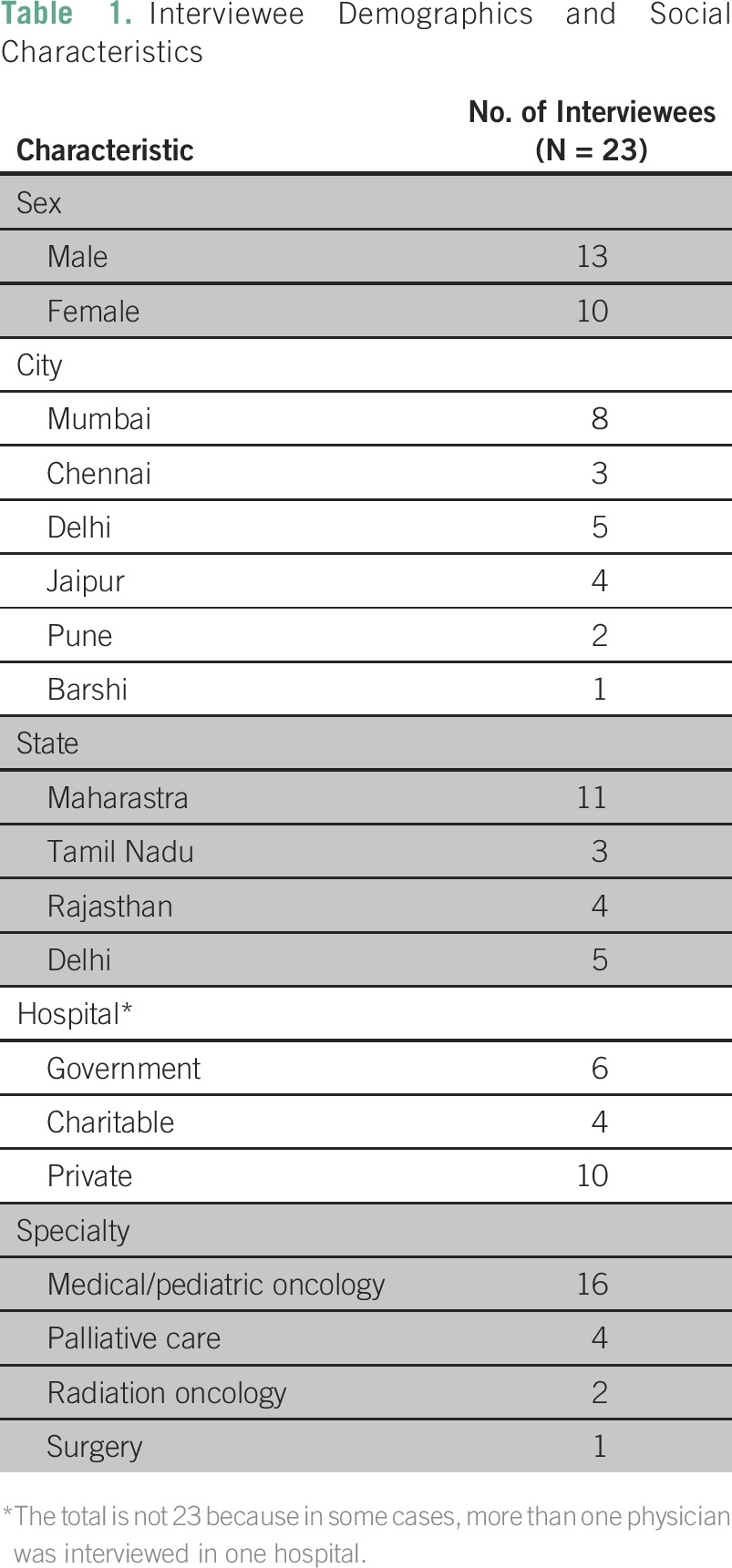
Interviewee Demographics and Social Characteristics

### Financial Issues

Financial issues dominated the discussions at all levels (family, hospitals, and government). Despite the existence of a public health care system, the majority (71%) of health care expenditure is out of pocket at the time of service.^18,19^ Morphine is inexpensive because opium is produced and manufactured locally, but indirect costs represent a heavy burden on families: “If they come from a remote village, for them to come back and follow up [to modify morphine doses or receive a new prescription] is next to impossible. They’ll mortgage land” (interviewee number 19 [Int#19]). At the hospital level, financial issues affect patient care, particularly in public hospitals:

The payment to the doctors...is not very attractive. The nurses come here, get some training, and they want to push off to a better hospital. So this is also one reason that it becomes difficult for us to train them. (Int#19)

Many physicians maintain private practice or move to private hospitals with attractive salaries.

### Lack of Education

Pain management and palliative care have only recently been introduced into medical college curricula. “If you could introduce this [supportive care training] as a basic training for...doctors who are already in service, I think it will make a big difference” (Int#23). Frequent rotations through various departments and limited use of opioids limit opportunities for nurses and junior physicians to gain experience in pain management. Many interviewees believed that they could not trust their nurses to properly monitor patients as a result of lack of time and training. As one physician explained, “[Intravenous] morphine actually requires a lot of monitoring. Sisters [nurses] are not trained here for IV morphine monitoring. That’s why I avoid it” (Int#4). The fear of addiction or severe adverse effects, particularly respiratory depression, seemed to increase with limited experience. The use of visual or numeric scales to monitor pain is sporadic and limited to the most affluent institutions (ie, private hospitals).

### Lack of Awareness

Lack of awareness is widespread among physicians and parents: “I really don’t have too much of the trouble of pain” (Int#14).

Most of our pain episodes are related to the initial either tumor mass or ALL [acute lymphoblastic leukemia] fresh diagnosis having body ache. We undertreat pain very significantly. I realize it is somehow not part of the process of assessing a patient. (Int#1)

In most public hospitals, invasive procedures are performed without conscious sedation, and postoperative pain is managed on patient request or by using standardized protocols without the monitoring and tailoring of treatment. Parents are not educated to recognize signs of pain:

It is not a question of lack of time, rather lack of awareness....They very rarely complain about things. A child will be irritable and cranky, and they think it is normal because of the overall disease rather than pain as such. They don’t know that painkillers are available and often associate morphine with terminal disease and death and refuse it. (Int#1)

### Lack of Human Resources

“There is always a huge mismatch between the numbers and the caregivers, and that is the chief reason why our quality is not as it should be” (Int#21). According to The World Bank, the number of physicians per 1,000 patients was 0.7 in 2012 (compared with 2.1 in Canada).^20^ Manpower is particularly scarce in public hospitals, and this (together with financial issues) creates the gap in the quality of care between private and public facilities. Such a shortage of health care providers and consequent shortage of time dedicated to each patient imposes prioritization: Physicians rarely ask about pain, and patients prefer talking about more important subjects, such as treatment or prognosis. “They think that if I will talk more about pain, they will just treat pain and they will not be able to give treatment for my child’s disease” (Int#23). Manpower shortage dictates the organization of work. “The staff population is so moving around that someone who is starting to learn the process usually leaves the department because his time here is over” (Int#1). “[Nurses] usually last a longer period than the residents in a particular ward. Over that period of time, they end up getting a significant expertise. My focus is to try to empower the nurses” (Int#1). “The trend here is not to let the nurses take their own decision...the nurses should have more authority to change the dose” (Int#1). “Nurses are more meant to take orders...they are too busy” (Int#3). In public hospital inpatient units, one nurse may take care of > 50 patients. The huge workload leads to undue delays in management and therapeutic adjustment and makes the use of some drugs (eg, intravenous morphine) unsuitable. In the smaller centers, the situation is even gloomier: “We don’t have competent doctors in the district areas and rural areas” (Int#18).

### Poor Availability of Opioids

A broad choice of opioids in various formulations are available in the country, including morphine, pentazocine, meperidine, and fentanyl. However, “the licensing is so difficult that people don’t apply. Unless you have all five licenses, no institute can buy and dispense morphine” (Int#19). As a result, opioids are available only in a few pharmacies and hospitals, and the dispensation procedure is tortuous. Pharmacists and physicians are afraid of being considered accountable for misuse or diversion and the heavy legal implications: “Sisters have to take specific precautions with respect to this, and I am not very comfortable” (Int#5). “When the drug controller comes, you have to account for even half a tablet. In a public hospital, nobody wants to take such responsibilities” (Int#17). Most institutions have access to only oral morphine and sometimes to fentanyl patches, which are expensive. The limited distribution network forces families to undergo long travels, which increases the financial burden. This also limits the possibility for physicians and nurses to gain experience in the use of opioids and thus generates a vicious cycle. Physicians sometimes have to prescribe inappropriate drugs, such as pethidine or crude opium tablets: “At times I ask my patients to have the crude opium” (Int#22).

### Successful Experiences

Despite the overwhelming difficulties, successful experiences were also identified. In a hospice in Chennai, a physician reported high-quality pain management: “[Nurses] are round the clock. We depend on them to tell us what is what….We have given them the freedom to escalate or give the [as needed] dosage” (Int#17). Adequate pain control is achieved in most situations with oral morphine and plays an important educational role: “There was a stigma attached with the hospice that whoever enters it dies....We are consciously encouraging admitting patients who are not so sick and sending them home several times” (Int#17).

## DISCUSSION

The main findings of this study are summarized in the words of one interviewee: “The first major problem which I would rank is poverty. Second thing would be illiteracy. The third thing would be the sheer numbers, and this automatically translates into lack of trained manpower. The last would be availability of drugs” (Int#21).

### Poverty

India adopted a public sector–led model of health care, but given inadequate investment from the government (< 1% of the gross domestic product is devoted to health care), the private system has expanded and now provides more than one half of medical care at unaffordable costs for uninsured people (the majority).^19^ The Planning Commission of India in 2010 recommended an increase in health expenditures to 2.5% of the gross domestic product to achieve universal health care by 2022.^21^ In addition, nontransmittable diseases must become a priority, although the agenda has so far been dominated by maternal health and infectious diseases.

### Illiteracy

Lack of education is reported in many studies.^22-24^ Indian physicians reported lack of education in pain control and palliative care as well as in medical and oncology education, particularly among nurses and general practitioners. “A nurse who is trained in this can do wonders” (Int#5). Some interviewees suggested the creation of ad hoc guidelines for junior physicians. Many programs have used the World Health Organization ladder^25-27^ and have included a training program for physicians and other operators (nurses and volunteers), often in more than one session (eg, yearly).^25^ In Uganda, a highly effective model was created by nurse practitioners as part of a palliative care plan, and because these nurses were only allowed to prescribe palliative care drugs, they also fulfilled the need for dedicated staff.^25^ These examples show how a change of approach to nursing and a long-established culture of subordinate relationship of nurses to physicians is possible. In India, recent initiatives (eg, the introduction of oncology and pain management in the undergraduate medical curriculum, creation of fellowships in palliative care, the formation of the Indian Association of Palliative Care, workshops on pediatric cancer targeted to family physicians) are likely to bring improvement.

### Sheer Numbers

The size of the country, the high percentage of population living in rural areas, and the scarcity of health care providers call for the development of a model of decentralized care. In rural settings in Uganda and Malaysia, programs that involve nurses trained and enabled to prescribe drugs for palliation^25,27^ are successful in reaching almost all patients with cancer in need of palliation. Traditional healers and even trained volunteers have proven to be useful health care providers.^6,25,28^ An example of this comes from the cancer registry of Barshi, the only rural cancer registry of India, in a remote village in Maharastra. Its nine employees visit > 500 villages regularly, educate the population about cancer, and identify individuals with symptoms and refer them to the hospital clinic or to camps held periodically where they can undergo diagnostic procedures. In this area, no general practitioner exists; the employees of the registry are the only health care providers.

### Availability of Drugs

Poor availability of opioids in India dates back to the late 1980s when the government issued the NDPSA to fight misuse and diversion of morphine. India is still a major world producer, manufacturer, and exporter of opium and opioids. Through judicial cases, physicians and nongovernmental organizations have tried to liberalize the use of medical opium.^29-32^ A recent amendment of the NDPSA to reduce the number of licenses needed has effectively improved the availability of opioids in 13 states, but more is needed. The state of Kerala is unique in the Indian landscape because palliative care services were started in the late 1990s by volunteers who created the Neighborhood Network in Palliative Care, and this initiative was followed by a state policy (Pain and Palliative Care policy) in 2008 that was implemented by the State National Rural Health Mission. This project focuses on primary level care, including home care and free delivery of medications and devices. Key to the success has been an awareness campaign from the media, which has resulted in strong community support, integration within the government health care system, and capacity building at the medical level.^33^ Relatively minor changes, such as the possibility to prescribe drugs for 1 to 3 months, an increase in the number of pharmacies that distribute opioids, and improved access to sustained-release formulations of morphine (now barely available), can translate into major improvements.^25,34,35^ Tables 2 and 3 summarize the contradictions of the Indian landscape and suggested solutions from the interviewees.

**Table 2:**
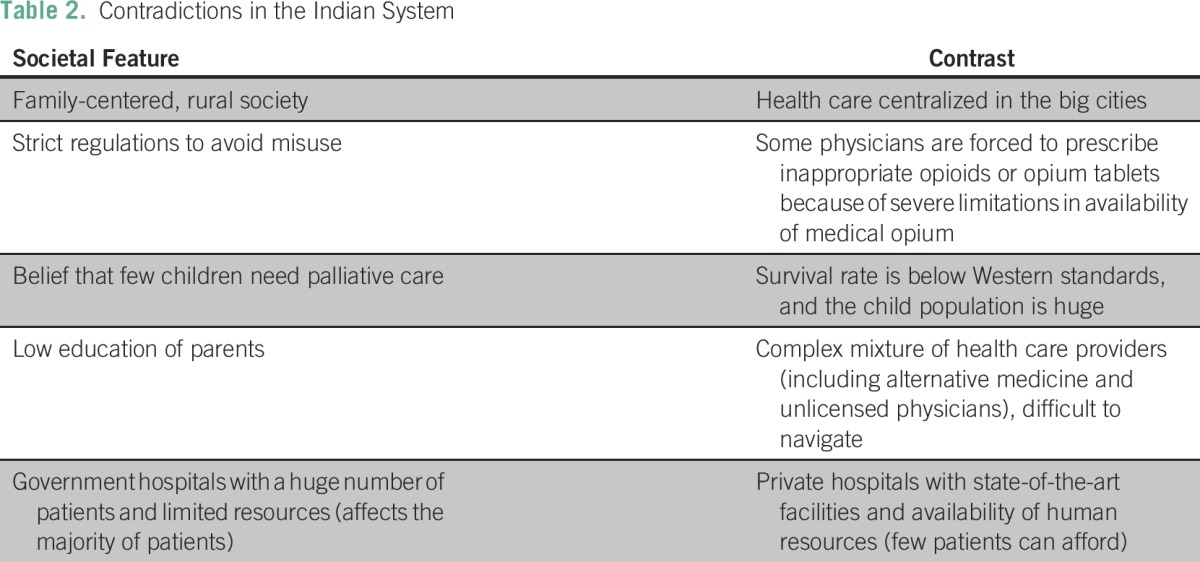
Contradictions in the Indian System

**Table 3:**
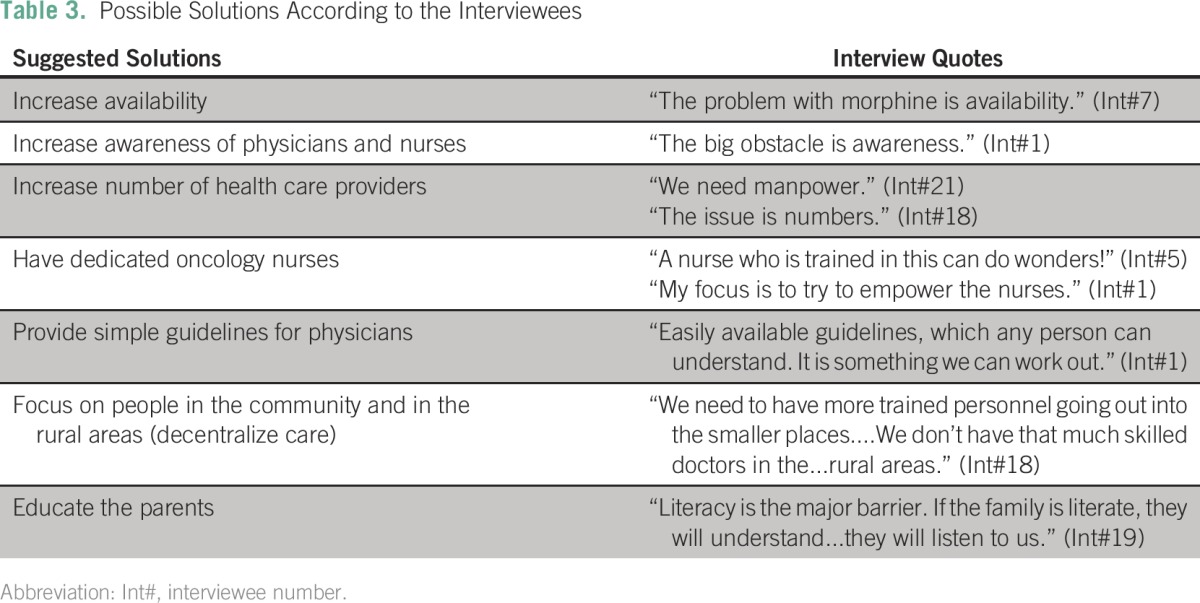
Possible Solutions According to the Interviewees

The issues described are not unique to pain control but represent major priorities for the development of health care in India. Is it still worth it to ask the question and look for obstacles to pain control in India? The interviewees challenged the study question itself. Two of the most senior physicians interviewed said that pain control is not a priority in India and identified lack of access to medical care, which leads to a missed diagnosis of approximately 80% of childhood cancer (“If you give me a million dollars, I would spend it on getting those 80% patients in rather than spending any dollar on pain management” [Int#9]) and infections (“Most important [problem in India] I would say [is] infection” [Int#25]) as the most common causes of childhood death.

Despite our efforts to choose a sample representative of the diversity of Indian reality, our choices led to multiple biases. We chose not to use translators but to interview only physicians, who are usually fluent in English. Because physicians appear to play a major role in pain management, we are confident that we got a comprehensive overview of the issues. In settings where nurses play an independent role, physicians and nurses have similar perspectives.^36,37^ Our choice may have led to an underestimation of patient-related cultural issues, which were only reported by physicians. We mostly explored the urban reality. Whereas 70% of the Indian population lives in rural areas,^3^ only one interview was conducted in a rural hospital, and three more were done in Jaipur, the only oncology referral center in Rajasthan, which highlights the issues experienced by the rural population. We interviewed only allopathic physicians. Because alternative medicine practitioners outnumber trained practitioners, the question of whether to involve them in pain control and palliative care is legitimate; however, this would have complicated the study beyond our intention and can be the object of a future investigation.

This study provides an overview of the barriers to opioid use in India and suggests areas for improvement. Physicians must advocate for pain management at all levels—government, health care provider, and even family—to improve the lives of children with cancer.
